# Seroprevalence of Brucellosis in Cattle, Sheep, and Goats in the Southeastern Region of Bangladesh

**DOI:** 10.1155/vmi/6843824

**Published:** 2026-08-04

**Authors:** F. M. Yasir Hasib, Md. Sirazul Islam, Chandan Nath, Md. Moktadir Billah Reza, Md. Masuduzzaman, Sharmin Chowdhury

**Affiliations:** ^1^ Department of Pathology and Parasitology, Faculty of Veterinary Medicine, Chattogram Veterinary and Animal Sciences University, Khulshi, Chattogram 4225, Bangladesh, cvasu.ac.bd; ^2^ Melbourne Veterinary School, Faculty of Science, The University of Melbourne, Parkville 3052, Victoria, Australia, unimelb.edu.au; ^3^ Department of Microbiology and Veterinary Public Health, Faculty of Veterinary Medicine, Chattogram Veterinary and Animal Sciences University, Khulshi, Chattogram 4225, Bangladesh, cvasu.ac.bd; ^4^ Department of Medicine and Surgery, Faculty of Veterinary Medicine, Chattogram Veterinary and Animal Sciences University, Khulshi, Chattogram 4225, Bangladesh, cvasu.ac.bd

**Keywords:** Bangladesh, brucellosis, epidemiology, seroprevalence

## Abstract

Brucellosis is one of the major bacterial zoonoses worldwide. Bangladesh is an endemically affected country with this neglected disease. This cross‐sectional study aimed to estimate the seroprevalence of brucellosis among cattle, goats, and sheep in the southeastern part of Bangladesh, covering Chattogram and Khagrachhari districts. A total of 1000 animals (cattle = 420, goat = 300, sheep = 280) were studied from July 2019 to June 2020 from a selected combination of 105 herds and households. First, all the samples were tested with the Rose Bengal plate test (RBPT). Later, RBPT‐positive samples and an equal number of randomly selected RBPT‐negative animals were tested using indirect enzyme‐linked immune sorbent assay (I‐ELISA). Overall true prevalence (TP) of brucellosis was 5.29% [95% confidence interval (CI): 0.80–9.77)], 12.86% (95%CI: 6.7021.29), and 13.40% (95%CI: 6.26–20.53), respectively, for the cattle, goat, and sheep of Chattogram, whereas the TP were 16.49% (95%CI: 8.57–24.40), 24.61% (95%CI: 14.92–34.29), and 16.30% (95%CI: 8.42–24.17), respectively, for the cattle, goat, and sheep of Khagrachhari. Notably, this study found that 67.24% (95%CI: 54.42–77.92) and 86.60% (95%CI: 68.56–104.63) of herds and households, respectively, in the Chattogram and Khagrachhari districts were positive for brucellosis for at least a single positive case. Agreement between the diagnostic tests RBPT and I‐ELISA was 86.90% (kappa agreement = 0.737) in this study. A multilevel multivariable logistic regression model depicts that prevalence was significantly higher in the Khagrachhari region for both cattle [odds ratio (OR) = 2.63 (95%CI: 1.28–5.42)] and goats (OR = 2.08; 95%CI: 1.15–3.75), whereas prevalence was significantly lower in aged individuals (OR = 0.54; 95%CI: 0.27–1.08). This study summarizes that this zoonotic disease in the study areas persisted at a considerably high level that might pose a substantial threat to public health. The findings will be a great help for the corresponding authority to take necessary intervention steps in controlling this zoonotic disease effectively.

## 1. Introduction

Brucellosis is a zoonotic disease affecting humans and numerous animal species such as cattle, buffalo, sheep, goats, dogs, cats, and pigs [1]. This disease, also known as Bang’s disease, undulant fever, Malta fever, or Mediterranean fever, is caused by Gram‐negative intracellular harmful bacteria under the genus *Brucella* [2]. Several species were found responsible for brucellosis, such as *B. abortus*, *B. melitensis*, *B. suis*, *B. canis*, *B. ovis*, and *B. neotomae* [3]. This zoonotic disease can be spread from infected animals to humans through direct contact with susceptible animal secretions or ingestion of unpasteurized milk, milk products, or parboiled meat [4, 5]. Brucellosis is an endemic disease in many parts of the world, including Bangladesh, after being identified first in the 1880s [6]. Reproductive abnormalities, particularly late abortion, retained placenta, and orchitis, are major clinical signs of brucellosis in affected animals [7–9]. But brucellosis is not a common direct cause of abortion in humans; however, it may be significantly associated with abortion cases and should be considered as a potential risk factor in endemic regions like Bangladesh [7–9]. According to the Department of Livestock Services (DLS) of Bangladesh, the populations of cattle, sheep, and goats were 25.1 million, 3.9 million, and 27.2 million in fiscal year 2024–2025, respectively [10]. Economic loss due to brucellosis is significantly high, affecting the socioeconomic condition of the farmers. There were different factors identified as risk factors for *Brucella* infection, for example, sex, age, breed, grazing system, herd size, and hybrid rearing system [11]. Moreover, the infection rate in natural insemination was higher than in artificial insemination [12]. Data related to the prevalence of the brucellosis infection were scarce in Bangladesh, but approximate prevalence was estimated to be around 2%–18% for humans, 5%–16% for cattle, 0%–4.8% in sheep, and 0.7%–14.6% in goats [13–17]. Brucellosis can be diagnosed by different procedures for both humans and animals, including serological tests Rose Bengal plate test (RBPT), slow agglutination test (SAT), tube agglutination test (TAT), standard tube agglutination test (STAT), mercaptoethanol test or ELISA (indirect, competitive, Avidin‐Biotin), fluorescent antibody test (FAT), or molecular techniques like polymerase chain reaction (PCR) and real‐time PCR [18, 19]. However, the sensitivity of the diagnostic tests varies among the methods [20, 21].

This study was designed to address the lack of knowledge on the seroprevalence of brucellosis in the study area, particularly in sheep and goats in the hilly region of Bangladesh, and to update data on infection rates. Through this investigation, we can compare the seroprevalence with associated risk factors in the study area, which may act as a reference point for the prevention and control of brucellosis in the selected regions.

## 2. Methods

### 2.1. Ethical Approval

Ethical approval was obtained from the Institutional Ethical Approval Committee of Chattogram Veterinary and Animal Sciences University (CVASU), Bangladesh, with the code of the approval [(CVASU/Dir(R&E) EC/2020/169/9)].

### 2.2. Study Area and Experimental Design

This cross‐sectional study was conducted from July 2019 to June 2020 in two southeastern districts of Bangladesh, namely, Chattogram (22.3569 N, 91.7832 E) and Khagrachhari (23.1108 N, 91.9906 E). Two districts were selected purposively to investigate the discrepancy in the ubiquity of brucellosis between the plain lands (Chattogram) with the hilly Area (Khagrachhari).

Sample size was calculated using the formula *n* = *Z* ^2^ *P*(1 − *p*)/*d* ^2^, where *Z* is the Z‐score corresponding to the desired confidence level, *p* is the expected prevalence, and *d* is the precision (margin of error). Assuming a 95% confidence level (*Z* = 1.96), a precision of 5% (*d* = 0.05), and an expected prevalence of 10% (*p* = 0.10) for all three species, the minimum required sample size was estimated to be 138.

Data were collected using a standardized, pretested questionnaire designed to gather information from farm record sheets and through direct, face‐to‐face interviews with farm owners for cattle sampling and household owners for sheep and goat sampling. In total, 105 farms and households were included in this investigation in consultation with local veterinary professionals. Verbal consent was obtained from the owners for metadata collection and its subsequent use in scientific publications. For cattle sampling, farms were randomly selected from the Department of Livestock Services (DLS)–enlisted farms, with a minimum cutoff of 10 cattle per farm. Within each selected farm, individual animals were randomly chosen as sampling units. However, for the sampling of goats and sheep, households were selected using a convenience sampling approach, prioritizing those with larger animal populations and longer histories of animal rearing. It is also important to note that convenience sampling was performed due to the fact that no sampling frame was available for households with animal populations in the study area. Both herd‐level and animal‐level epidemiological data were collected for analysis. The animals were categorized based on age, breed, sex, insemination type, knowledge of brucellosis, abortion history, and production type. A total of 1000 animals were surveyed, comprising 420 cattle, 300 goats, and 280 sheep. From Chattogram, 300, 124, and 150 blood samples were collected from cattle, sheep, and goats, respectively, while from Khagrachhari, 120, 156, and 150 blood samples were collected from the same species.

### 2.3. Blood Collection and RBPT Test

Blood samples were aseptically drawn using 4‐mL plain vacutainers from the jugular vein of sheep and goats, and from either the tail vein or jugular vein of cattle. All blood samples were immediately placed in ice packs for transportation to the laboratory. Serum was separated from the whole blood samples using a standard procedure. Each serum sample was transferred into a 1‐mL Eppendorf tube and stored at −80°C for further analysis to maintain stability and prevent degradation. The samples were later tested using the Rose Bengal dye (ID Vet Grabels, France, LOT: RSA‐RB‐025) (Screen Brucellosis Serum Indirect Multi‐Species kit [Grabels, France]), following the manufacturer’s instructions. Equal volumes of serum and reagent were mixed on a microscopic slide, and the mixture was gently agitated. The appearance of agglutination, visible to the naked eye, was considered a positive reaction.

### 2.4. I‐ELISA Test

Three hundred samples were examined through the I‐ELISA test, including all the RBPT‐positive samples, and the remaining samples were selected randomly from the RBPT‐negative animals with equal ratios from each animal species. I‐ELISA examination was performed according to the manufacturer’s manual, where the procedure ends with adding the stop solution. ID Screen Brucellosis Serum Indirect Multi‐Species kit (Grabels, France, LOT: 690‐011) was used for this serological examination. The optical density (OD) value was measured to interpret the result in a digital ELISA reader (Convergys EL‐Reader 96X, Coelbe, Germany). A sample with a positive ratio (S/P) greater than or equal to 120% was considered positive for Brucella infection. This I‐ELISA kit can diagnose seropositivity of multispecies *Brucella* organisms such as *B. abortus*, *B. melitensis*, *B. suis*, and *B. ovis*.

### 2.5. Statistical Analysis

Univariable and multivariable logistic regression analyses were conducted using STATA‐IC 13 to identify potential risk factors, with a 95% confidence interval (CI) applied. A household was classified as positive if at least one positive sample was detected within it. Herd prevalence was calculated as the proportion of positive households relative to the total number of households. Risk factor analysis at the individual animal level and herd level was conducted using a random‐effect model where farm and area were included as random effects, respectively. In multivariable logistic regression analysis, the backward elimination technique was employed to reach the final model. A single variable was eliminated from the models at a time with the highest *p* − *v* *a* *l* *u* *e*, and the likelihood ratio test (LR test) was conducted after running each model to find the best‐fit model. A *p* − *v* *a* *l* *u* *e* ≤ 0.05 was considered significant in the LR test, where the null hypothesis was that the model fits the data equally well. In univariable and multivariable logistic regression analysis, variables with a *p*−value ≤ 0.05 were considered statistically significant risk factors. Multicollinearity among the variables was checked in the logistic regression models by temporarily treating the binary variables as continuous ones and a variance inflation factor (VIF) > 10 was considered problematic multicollinearity among factors. Furthermore, the adjusted prevalence was defined as the true prevalence (TP), corrected using the test kit’s performance parameters (sensitivity 98% and specificity 98%) as mentioned on the manufacturer’s package. TP was calculated using the following formula: TP = ((*A* *P* + *S* *p* − 1)/(*S* *e* + *S* *p* − 1))*X*100. To test the agreement between tests (RBPT and I‐ELISA), kappa statistics was employed where kappa value was interpreted as: poor (*κ* = 0), slight (0.01 < *κ* < 0.20), fair (0.21 < *κ* < 0.40), moderate (0.41 < *κ* < 0.60), almost perfect (0.61 < *κ* < 0.80), and excellent (0.81 < *κ* < 1.00) [22]. Here, I‐ELISA was considered the gold standard/better test as it has better sensitivity and specificity compared to RBPT according to the manufacturer’s manual. Apparent prevalence (AP) of brucellosis at the herd level in the study areas was visualized in digital maps using ArcGIS Version 3.3.

## 3. Results

### 3.1. Prevalence of Brucellosis Diagnosed by RBPT Test

The AP and TP were calculated from the RBPT test as depicted in Table 1. In most cases, the prevalence was higher in Khagrachhari than in Chattogram in terms of individual level and herd level. Point locations of the households with animals selected for this study, along with individual‐level AP, were visualized in digital maps in Figure 1a and Figure 1b for the Chattogram and Khagrachhari districts. In Chattogram, the TP was 5.29, 12.86, and 13.40; in Khagrachhari, the rates were 16.49, 24.61, and 16.30 for cattle, goats, and sheep, respectively. 68.08% and 86.60% of herds were chronologically positive (TP) for brucellosis in Chattogram and Khagrachhari districts.

**TABLE 1:** Apparent prevalence and estimated true prevalence of brucellosis identified by RBPT at individual and herd levels.

Species	Gender	Chattogram				Khagrachhari			
		Total	Positive	AP (95% CI)	TP (95% CI)	Total	Positive	AP (95% CI)	TP (95% CI)
Cattle	Female	300	20	6.67 (4.35–9.48)	5.29 (0.80–9.77)	115	19	16.52 (10.84–24.37)	15.48 (7.81–23.14)
	Male	0	0	0	0	05	2	40.00 (11.76–76.93)	39.83 (28.92–51.07)
	Total	300	20	6.67 (4.35–9.48)	5.29 (0.80–9.77)	120	21	17.50 (11.74–25.28)	16.49 (8.57–24.40)

Goat	Female	133	18	13.53 (8.73–20.38)	12.38 (5.42–19.14)	134	34	25.37 (18.76–33.36)	24.66 (14.97–34.34)
	Male	17	03	17.65 (6.19–41.03)	16.65 (8.90–24.39)	16	04	25.00 (10.18–49.50)	24.27 (14.94–33.59)
	Total	150	21	14.00 (9.34–20.46)	12.86 (6.70–21.29)	150	38	25.33 (19.05–32.85)	24.61 (14.92–34.29)

Sheep	Female	111	16	14.41 (9.07–22.14)	13.29 (6.19–20.38)	139	25	17.99 (12.49–25.20)	17.00 (8.96–25.03)
	Male	13	02	15.38 (4.32–42.23)	14.30 (7.18–21.41)	17	02	11.76 (3.28–34.34)	10.54 (4.38–16.69)
	Total	124	18	14.52 (9.38–21.78)	13.40 (6.26–20.53)	156	27	17.31 (12.18–24.01)	16.30 (8.42–24.17)

Herd	Total	58	39	67.24 (54.42–77.92)	68.08 (52.06–84.09)	47	40	85.10 (72.31–92.59)	86.60 (68.56–104.63)

Abbreviations: AP = apparent prevalence, CI = confidence interval, TP = true prevalence.

FIGURE 1(a): Point location of the farms along with individual‐level seroprevalence of brucellosis in Chattogram district. (b): Point location of the farms along with individual‐level seroprevalence of brucellosis in Khagrachhari district.

**Figure:**
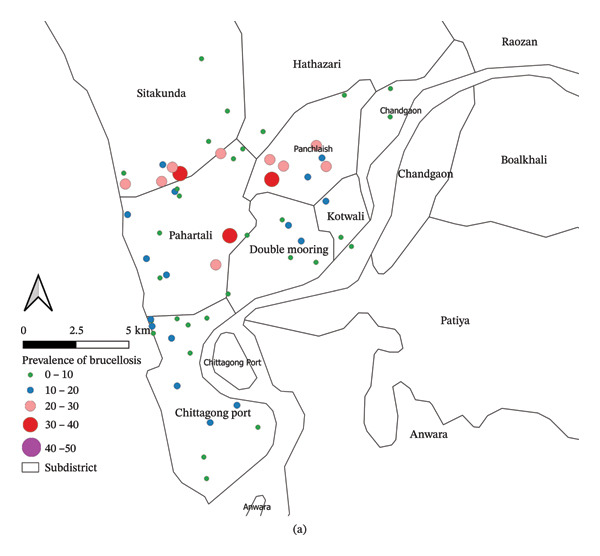

**Figure:**
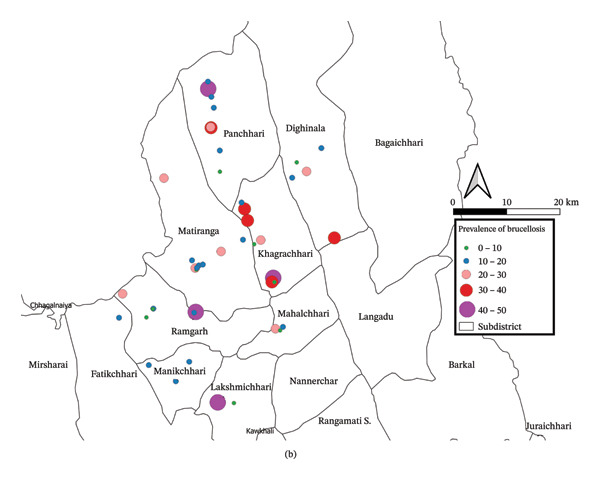

### 3.2. Risk Factor Analysis of Brucellosis Diagnosed by RBPT Test at Individual Animal Level

Individual‐level risk factors were analyzed by both the univariate and multivariate logistic regression models, and the results are presented in Table 2 and Table 3, respectively. In univariable analysis for cattle, area and age were two risk factors that showed significant association, whereas for goats, only area was identified as a risk factor. But no individual‐level risk factors were significant for sheep in univariable analysis. On the other hand, the multilevel multivariable logistic regression model depicted that prevalence was significantly higher in the Khagrachhari region for both cattle (odds ratio [OR] = 2.63; 95%CI = 1.28–5.42) and goats (OR = 2.08; 95%CI = 1.15–3.75).

**TABLE 2:** Potential risk/indicator factors associated with individual animal‐level brucellosis seropositivity diagnosed using RBPT based on a random‐effects model (farm as a random effect).

Variable	Level	*N* (animal)	Positive *N* (%)	OR	CI	*p*‐value
*Cattle*						
Area	Chattogram	300	20 (6.67)	1		0.002
	Khagrachhari	120	21 (17.50)	2.96	1.48–5.94	
Sex	Female	415	39 (9.40)	1		0.08
	Male	5	2 (40.0)	5.63	0.79–40.07	
Age	36 months	126	19 (15.08)	1		0.02
	> 36 months	294	22 (7.48)	0.46	0.23–0.92	
Breed	Local	68	9 (13.24)	1		0.33
	Cross	352	32 (9.09)	0.67	0.29–1.51	

*Goat*						
Area	Chattogram	150	21 (14.00)	1		0.01
	Khagrachhari	150	38 (25.33)	2.08	1.15–3.75	
Sex	Female	267	52 (19.48)	1		0.81
	Male	43	7 (21.21)	1.11	0.45–2.70	
Age	12 months	28	6 (21.43)	1		0.80
	> 12 months	272	53 (19.49)	0.88	0.34–2.29	
Breed	Local	0	—	—	—	—
	Cross	300	59 (19.67)	—	—	

*Sheep*						
Area	Chattogram	124	18 (14.52)	1		0.52
	Khagrachhari	156	27 (17.31)	1.23	0.64–2.35	
Sex	Female	250	41 (16.40)	1		0.66
	Male	30	4 (13.33)	0.78	0.25–2.36	
Age	12 months	87	9 (10.34)	1		0.08
	> 12 months	193	36 (18.65)	1.98	0.91–4.33	
Breed	Local	130	17 (13.08)	1		0.20
	Cross	150	28 (18.67)	1.52	0.79–2.93	

**TABLE 3:** Final multilevel multivariable logistic regression model (farm as a random effect) showing the significant (*p* < 0.05) association of different animal‐level factors with the presence of brucellosis diagnosed using RBPT.

Variable	Level	OR	95% CI	*p*‐value
*Cattle*				
Area	Chattogram	1		0.008
	Khagrachhari	2.63	1.28–5.42	
Age	36 months	1		0.08
	> 36 months	0.54	0.27–1.08	

*Goat*				
Area	Chattogram	1		0.01
	Khagrachhari	2.08	1.15–3.75	

*Sheep*				
Age	12 months	1		0.08
	> 12 months	1.98	0.91–4.33	

### 3.3. Risk Factor Analysis of Brucellosis Diagnosed by RBPT Test at Herd Level

All the herd‐level risk factors were nonsignificant in the univariable regression analysis in this study (Table 4). However, an increased OR for the semi‐intensive and extensive housing systems was observed. Besides, natural insemination, history of abortion, mixed farming, and knowledge about the disease did not show any significant association with the outcome.

**TABLE 4:** Potential risk/indicator factors associated with herd‐level brucellosis seropositivity diagnosed using RBPT based on a random‐effects model (area as a random effect).

Variable	Level	*N* (animal number)	Positive *N* (%)	OR	CI	*p*‐value
Farm type	Intensive	26	16 (61.54)	1		
	Semi‐intensive	56	45 (80.36)	2.27	0.69–7.44	0.17
	Extensive	23	17 (73.91)	1.59	0.41–6.05	0.49

Breeding type	AI	62	46 (74.19)	1		0.78
	Natural	42	32 (74.42)	1.13	0.45–2.86	

Abortion (last 12 months)	No	75	53 (70.67)	1		0.24
	Yes	30	25 (83.33)	1.91	0.63–5.77	

Mixed farming	No	16	10 (62.50)	1		0.39
	Yes	89	68 (76.40)	1.66	0.51–5.40	

Knowledge on brucellosis	No	92	69 (75.00)	1		0.77
	Yes	13	9 (69.23)	0.82	0.22–3.01	

### 3.4. Comparison Between RBPT and I‐ELISA Tests

Agreement between tests was 86.90%, with the kappa value calculated being 0.737 (almost perfect). The sensitivity and specificity of RBPT were 81.66% (CI 74.99%–87.18%) and 94.21% (CI 88.44%–97.64%) when compared with I‐ELISA (considered as the gold standard/better test). Moreover, positive predictive value (PPV) and negative predictive value (NPV) were 82.47% (95%CI: 69.55%–90.65%) and 93.91% (95%CI: 91.79%–95.50%) for RBPT.

## 4. Discussion

Brucellosis in Bangladesh is considered as endemic disease affecting small and large ruminants [23]. Both production and economic impacts have been observed in affected animals. Very few studies all around Bangladesh were conducted to know the actual prevalence, particularly in the hilly areas, where an underdeveloped management system is supposed to cause a more significant majority of this neglected zoonosis.

This research found a notably higher herd‐level seroprevalence of brucellosis estimated as 67.24% and 85.10% in Chattogram and Khagrachhari districts, respectively. Seropositivity among the herds ranged from 5 to 20%, irrespective of species, in a previous study [13]; the difference might be due to the definition set up to consider a herd positive OR diagnostic tests performed OR sampling strategy. In Chattogram, previous studies reported that the individual animal‐level seroprevalence among ruminants ranged from 5 to 15% for cattle, sheep, or goats [14, 15, 24]. Our estimates almost matched the previous findings from Chattogram and in regions of South Asia such as India and Pakistan [25–28]. In the Chattogram region, we observed that cattle were less susceptible compared to goats and sheep, and in Khagrachhari, cattle and sheep had nearly similar seroprevalence, whereas goats had the highest. Previous studies also reported higher seroprevalence in goats compared to cattle and sheep [29]. In Bangladesh, goats are generally reared in an extensive system, which might facilitate inter‐ and intraspecies transmission. Moreover, screening and interventions are less commonly practiced in goats compared to cattle. The increased seroprevalence in goats in both study areas might indicate the risk of human transmission via goat milk and direct contact, and therefore, public health policy should take this small ruminant into account, which is often neglected.

On the other hand, higher herd‐level and individual animal‐level prevalence was observed in the Khagrachhari district compared to Chattogram. No previous studies on this topic have been conducted in the hill tracts of Bangladesh, including Khagrachhari district. The increasing trend in seropositivity in the Khagrachhari region might be due to area‐specific factors like differences in farming management systems and inadequate awareness about the disease, which might be attributed to insufficient extension services in the area. Most of the areas of the hill tracts in Bangladesh are remotely located, and farmers of these areas might have less access to the treatment and extension services provided by the government livestock department. Moreover, the management system of hilly areas might also be very different from the plain land. To understand the actual reason behind this discrepancy between plain and hilly regions, an extended study should be conducted including more hilly districts of Bangladesh focusing on farm management and husbandry‐related factors.

Though previous studies identified semi‐intensive and extensive rearing systems, natural insemination, and lower knowledge about the disease as potential risk factors for a high prevalence of brucellosis, our study did not find evidence [5, 28, 30, 31]. However, we noticed that the semi‐intensive and extensive farming systems have a higher OR than intensive farming. Previous studies speculated that extensive farming experienced a higher prevalence rate compared to other farming systems might be due to a higher chance of direct contact between animals of different herds and subsequent disease transmission [32–35]. Though we did not find statistically significant evidence, we can hypothesize from observing a higher OR that it could be a potential risk factor and should be tested in an extended study.

Similarly, a history of abortion in the herd was associated with a higher chance of having a positive brucellosis status of the herd, similar to the findings of the peers. Moreover, mixed farming, small and large ruminants rearing together, appeared to be more likely to be infected (higher OR) with this zoonosis, as most of the causal agents for brucellosis can infect cross‐species [36–39]. None of the above‐mentioned herd‐level variables showed a statistically significant association with the outcome in our study. Failure to find a significant association, when in reality there might be an association (Type II error), might be attributed to different factors like inadequate sample size and measurement error. A sample size of 105 herds and households in our study might have contributed to achieving a nonsignificant association. Also, if the difference between the variables is very small, a large sample size might be necessary to get a significant association.

The sensitivity and specificity of RBPT found in this study were analogous to the previous studies, such as Rahman et al., reported 80%–82% and 98%–99% sensitivity and specificity, respectively, depending upon species. Islam et al. found 87% and 99% sensitivity and specificity, respectively [13, 15]. A minor difference in results might be due to differences in study settings, such as using kits and reagents from a different manufacturer. The kappa values of this study closely matched those of previous research that evaluated these two diagnostic tests. Therefore, it can be suggested that these two cheap and efficient methods of diagnosis can be used for screening purposes in Bangladesh.

Despite taking precautions during the design and analysis stages, the study might inherit some limitations. The use of convenience sampling in household selection might have introduced selection bias and therefore should be taken into account when interpreting the results. Moreover, the herd‐level prevalence was found remarkably high in both study areas, which might be because of the strategy taken to consider a herd positive for brucellosis (a herd considered positive when at least one animal is found positive in the herd). However, it also indicates that brucella‐positive animals are not concentrated in a few farms, and therefore, an extensive control strategy should be implemented in the area.

The presence of seropositivity in three different domestic animal species in plain and hilly areas of Bangladesh highlights the magnitude of transmission of the disease and the possibility of spillover in other species, including humans, in the reported areas. However, the identification of active infection and specific *Brucella* species using molecular techniques in different animal species and humans might be necessary to formulate effective prevention and control measures. As a zoonotic disease, we suggest that public health officials take necessary actions to inform the local people about the condition and proper investigation and diagnosis of every case of abortion in the region with care. We also recommend prospective studies using thorough molecular methods for confirmatory diagnosis of this disease in the study area with the collaboration of one health specialists so that overall improvement in public and animal health will be easier to achieve.

### 4.1. Limitations

The present study utilized the manufacturer‐reported sensitivity and specificity of the diagnostic kit. These parameters may differ under field conditions due to variations in sample quality, environmental factors, and testing procedures.

## 5. Conclusions

This study demonstrated a notable seroprevalence of brucellosis in food animals, particularly in small ruminants in the hilly region of Bangladesh, that is often underreported. The potential for economic losses and, most importantly, the zoonotic significance of the disease necessitate urgent intervention strategies to effectively control and eliminate brucellosis from livestock populations in Bangladesh.

**Nomenclature**
AP: Apparent prevalence
CI: Confidence interval
FAT: Fluorescent antibody test
I‐ELISA: Indirect enzyme‐linked immune sorbent assay
NPV: Negative predictive value
OR: Odds ratio
OD: Optical density
PCR: Polymerase chain reaction
PPV: Positive predictive value
RBPT: Rose Bengal plate test
SAT: Slow agglutination test
STAT: Standard tube agglutination test
TP: True prevalence
TAT: Tube agglutination test

## Author Contributions

F. M. Yasir Hasib: data curation, investigation, methodology, project administration, writing–original draft, and writing–review and editing; Md. Sirazul Islam: data curation, investigation, methodology, project administration, and writing–original draft; Chandan Nath: data curation, investigation, project administration, and methodology; Md. Moktadir Billah Reza: investigation; Md. Masuduzzaman: conceptualization, funding acquisition, resources, and writing–review; Sharmin Chowdhury: conceptualization, funding acquisition, methodology, data visualization, project administration, supervision, validation, and writing–review and editing.

## Funding

The authors would like to acknowledge the Ministry of Science and Technology, Bangladesh [NST fellowship for the fiscal year 2019–2020 (Serial number # 2735)] and the Chattogram Veterinary and Animal Sciences University (CVASU), Bangladesh, for the funding of this research.

## Ethics Statement

The study was approved by the institutional ethics committee of Chattogram Veterinary and Animal Sciences University (CVASU), Bangladesh, with the code of the approval [(CVASU/Dir(R&E) EC/2020/169/9)].

## Conflicts of Interest

The authors declare no conflicts of interest.

## Data Availability

The datasets presented in this study can be retrieved from the corresponding author upon reasonable request.
